# Science, Regulation, and Precedent for Genetically Modified Insects

**DOI:** 10.1371/journal.pntd.0001504

**Published:** 2012-01-31

**Authors:** John D. Mumford

**Affiliations:** Centre for Environmental Policy, Imperial College London, Silwood Park Campus, Ascot, United Kingdom; Liverpool School of Tropical Medicine, United Kingdom

Questions have been raised about the transparency and scientific quality of regulatory processes applied in the first open field releases of genetically modified (GM) insects, and there is concern that inappropriate precedents have been set [1], particularly through generic risk assessments covering multiple species and technologies [2]. The GM insects tested in the field so far are intended for sterile insect release programs against a major pest of cotton (pink bollworm, *Pectinophora gossypiella*) in the United States [3] and against *Aedes aegypti*, the mosquito vector of dengue fever, in the Cayman Islands [4], Malaysia [5], and Brazil [6]. In the pink bollworm release, the GM insects were sterilised by radiation and the GM trait was a heritable red marker colour that allowed discrimination from wild types. In the *Aedes aegypti* releases, the mosquitoes carried an inserted inherited dominant lethality trait that causes late larval stage mortality in offspring of homozygous and heterozygous individuals, as well as the colour marker trait to provide an essential discrimination from wild types. For control programs, insects with new genetic properties could be introduced either in large numbers in self-limiting populations or in smaller numbers in self-replicating populations for a specific beneficial purpose [7], [8], but releases have so far only involved the self-limiting category.

The release of GM insects into the environment poses two broad risk issues. There are potential environmental risks associated with the introduction of large numbers of any selected mass-reared population, in many cases of an alien species, that can interact with both the wild population of that species and other, non-target organisms, including humans. There are also specific risks related to the GM technologies introduced into the process, which may also interact with the broader environment through particular expressed traits, such as metabolic products, or through fitness differences compared to untransformed insects. In terms of mass release risks, we should expect GM insects to be regulated in a way that is similar to that applied to the widespread release of conventional beneficial insects, for which there are already international guidelines [9]. Given the potential for natural or assisted spread from insect releases, for both the intended organisms and any secondary pathogens, parasites, etc., this may need regional or international decisions (an example was the beneficial parasite of the cassava mealybug in Africa [10]). While such assessments should be carried out on a case-by-case basis, this process can build on the vast experience of new organism introductions that have already been made.

National regulations, while not consistent in operation across all countries, determine the conditions under which non-GM insects can be released, and examples of such releases are common from all continents. The release of GM insects is, in principle, a similar activity. There is not yet any widely accepted specific guidance for GM insect releases. There is a regional standard in North America for the movement and confined field release of transgenic insects [11], while international guidance is still in preparation on open field release of GM mosquitoes [12]. The Cartagena Protocol on Biosafety offers broad principles on risk assessment for GM organisms [13], but no specific guidance for GM insects.

Billions of mass-reared insects, which are not GM, are released into the wild each week around the world. Many are non-native pest species reared, irradiated, and distributed in sterile insect release programs [14]. In addition to sterile releases, fertile biological control agents, including insects, have also been released, so far in over 7,000 cases [15]. They are released in large numbers in continuous augmentative control programs and in smaller numbers for long-term self-replication to suppress pest species. Using a similar approach, mosquitoes infected with strains of *Wolbachia* bacteria intended to reduce their ability to transmit dengue have been released in the wild to establish self-replicating populations of infected mosquitoes [16]. Millions of pollinators are released on a regular basis in both protected and field crops, often imported from abroad [17].

The majority of these releases have been widely accepted as beneficial, although some have failed and some risks are recognised as constraints for these uses [14], [15], [17], [18]. As in the case of GM insect technologies, biological control scientists are calling for proper adherence to national regulations and international standards as they continue to search for and release new agents [15]. Pollination with imported pollinators is under review [17] due to declines in native pollinators in many countries. Some sterile insect procedures have begun to be standardised to assure quality and reduce risks [19]. A common standard for risk assessment procedures for mass-released insects may be helpful, given the scale of worldwide releases.

The appropriateness of generic environmental risk assessment has been questioned [1]. It may be more appropriate to consider generic risk factors, rather than to assess the risk of technically unrelated GM applications in a range of unrelated species. For example, there may be some aspects of risk that are genuinely generic, and where examples from other species and relevant applications can be useful. A generic risk in releasing GM insects with inherited lethality traits is whether sterile insect technique (SIT) will be successful with some proportion of females and “non-sterile” individuals present in the releases. The long history of radiation-induced sterile insect release in fruit flies, with conventional breeding of strains to develop genetic sexing so that “male only” releases can be made, gives some demonstration of the robustness of such programs [14]. Excessive radiation reduces fitness, so a compromise is made between fitness and sterility. As a result, typical sterility levels are around 95%–99% sterile [20] (the Cayman GM mosquitoes produced 96.5% lethality [4]). The sexing process is also not complete, yielding around 99% males at release for sterile Mediterranean fruit fly programmes [21] (the Cayman GM mosquitoes were 99.55% male [4]). Release programs work effectively and efficiently with these levels, although employing GM to achieve the same objectives could prove more efficient in many cases. The high level of sterility, albeit not perfect, and the predominance of released males over successive releases ensures that any small proportion of non-sterile insects or females do not have a significant impact on the overall control performance, particularly as wild populations diminish rapidly after releases start. Establishment of viable populations from SIT releases with small proportions of males and non-sterile individuals would be most likely to show up in preventative release programmes, where released insects have no direct intraspecific competition. With extensive surveillance, no populations of Medflies have established from released “male-only”, “sterile” Medflies in Florida or California, where preventative sterile insect release programs are underway [14]. In GM insect control under the SIT, even with some residual low levels of fertility and some females in the releases, similar results could be anticipated.

Fitness may also be a generic risk issue. GM pink bollworms were shown to be more fit, as measured by relative recapture in pheromone traps, than a conventional mass-reared strain, but this was attributed to the GM strain being more recently developed and reared under more relaxed conditions [3], and neither may be optimal. This emphasises the need to consider the whole rearing system, and not just the GM trait alone in risk assessments. GM mosquitoes released in the Caymans were less fit than wild types [4]. While relatively low fitness of mass-released insects is detrimental to performance, it is recognised as reducing the risk of unwanted persistence [8].

Have the regulatory processes in the United States, Cayman Islands, and Malaysia been an undue precedent for the decisions on GM insects elsewhere? In Brazil, the next release location, sterile male *A. aegypti* have been released in the field in the state of Bahia, starting in early 2011 [6], [22]. The release was approved by Comissão Técnica Nacional de Biossegurança (CTNBio) [23], the national regulator, after local review of the regulatory process, which had not previously addressed GM insects specifically. In Brazil, CTNBio was established to approve GM organism releases on the basis of an evaluation of plant, animal, and human health and environmental protection [24]. To achieve competence in assessing technical evidence, administrative processes, and public perceptions, the commission is composed of 27 members, including technical specialists and representatives of various responsible ministries. In the case of commercial release, a public hearing can be requested by interested parties, but CTNBio may agree to commercial confidentiality in relation to parts of some applications. While the process is not transparent, it should be publicly accountable. In terms of informed public consent, it was argued that there is a history of sterile fruit fly release in the area, from the local MoscaMed facility, where the GM mosquitoes are produced, and that people in the area may be more familiar with the concept of sterile insect release than in many places. Many examples of Brazilian television coverage can be seen on the Internet, where the releases have been set in the context of dengue control and have been indicated as transgenic. Many aspects of the process have been critically reviewed [22], though it is not clear that shortcomings are due to precedents from earlier cases.

The Malaysian decision to allow limited trial release of manually sexed male mosquitoes referred to the outcome of the Cayman Island trial, not the risk assessment for it [25]. It also allowed a step-wise release program in which each step of increasing assessed level of risk is dependent on assessment of earlier outcomes, so the decision is not based on broad acceptance of a generic approach. The decision was principally based on a risk assessment determining “quite low” risk, particularly taking into account risk mitigation, such as manual sexing. Risk assessment outcomes can be more formally described, for example, much effort has been made in this regard in the field of climate change [26], and acceptability can be pre-defined, as in the Australian gene technology risk analysis framework [27]. In the Australian definitions of risk acceptability, risk mitigation is taken into account, so even high intrinsic risks may be acceptable with feasible and effective mitigation.

In Europe, the European Food Safety Authority commissioned an extensive report on GM insect issues in 2010 [7] and is currently preparing guidance for GM insects within a broader context of GM animals [28], quite independent of any precedents elsewhere. The Advisory Committee on Releases to the Environment (ACRE) in the United Kingdom [29] recently considered the limitations of the current UK and European Union (EU) regulatory frameworks for GM insects and observed that, particularly in the case of GM vector control, there was a need for a more holistic approach than is currently provided by Directive 2001/18/EC, which focusses on environmental risks from new GM technology. ACRE considered there was a need for risks from a GM approach to be compared against the full range of risks associated with existing alternative approaches (such as insecticides) and the risks of inaction (inadequately controlled disease). It would be a significant shift to move from a forward-driven risk assessment process, in which ever diminishing marginal hazards are identified, to one addressing the probability that benefits will not exceed the combined sum of environmental, social, and other costs There are currently no international standards for assessing benefits from new organism releases [30].

An environmental risk assessment (ERA) for GM organisms in the EU should be done individually for each case [30]. The United States Department of Agriculture Environmental Impact Statement (USDA EIS) combining several species and technologies [2] states that it is aimed at providing a broad overview of impacts, and indicates procedures that the Animal and Plant Health Inspection Service (APHIS) would follow prior to the release of GM insects in which site- and application-specific risk characteristics would be considered. The USDA EIS envisages revisions of the broad overview of risks as new technologies are developed and new risks are identified. An EU GM organism ERA requires 1) data on the genetic modification and its mechanism in the species; 2) data on rearing, release, and the receiving environment; 3) data on the dynamic interactions of the GM insect and the environment; and 4) validated and detailed protocols for monitoring and control of the GM insect after release [30], [31]. A monitoring plan is needed to ensure that hazards not identified in the ERA can be detected as early as possible and remedial action taken. An emergency response plan that can prevent spread and further negative impacts if they occur is considered essential for risk communication to the public [30]. Responsibility is shared in the approval process, the regulator sets out the data required, the applicant provides the ERA to meet those requirements, and the regulator then assesses the validity and relevance of the use, methods, data, and release plans described.

Formally assessing the scientific quality of information for approvals for uncaged GM insect field trials [1] has merit. The Australian GM risk analysis framework [27] also includes a list of quality criteria for evidence, which includes reliability, transparency, robustness, etc., with examples of increasing levels of utility. This approach could be adapted further to assign semi-quantitative levels of confidence to the specific components of a risk assessment, as is done in some risk analyses for agricultural pests and non-native species [32]. This would allow the inevitable uncertainty to be explicitly addressed, where necessary. Explicit characterisation of uncertainty in specific risk components allows research priorities to be determined by developers and regulators.

How should potential benefits be considered? Risk assessment focusses on likelihoods and consequences of identified hazards, without direct consideration of benefits. However, the benefits of actions are often offset against the risks in determining how acceptable risks are in practical management decisions at a social or political level, where risk assessment and management can merge into benefit cost analysis [33]. If and when full-scale implementation of GM insects becomes a reality, risk assessment will be superseded by benefit-cost studies [34], where further issues related to appropriate measures of efficacy and the value of the threat that is controlled will arise and be debated, as the current risk assessments are. The Malaysian limited release decision takes note of socioeconomic drivers such as dengue deaths and medical costs, in addition to the risk assessment, which is already a step towards a benefit-cost approach to adoption. The Australian risk analysis framework notes that while benefits are not directly considered, they do affect perceptions of risk tolerance [27].

In the case of *Wolbachia*-infected mosquitoes in Australia, the regulatory problem formulation was defined through the endpoint hazard: will release of the infected mosquitoes “cause more harm”, considering adverse hazards and their likelihood over a 30-year horizon, than continuing to manage mosquitoes and dengue in a conventional way [35]? It was determined, through a combination of expert evidence and representative community participation, that the releases, which do not involve genetic modifications, had a negligible risk (the product of likelihoods and consequences) of causing more harm. One of the most significant components of this very small risk was that public perception would consider that the dengue problem was solved by the releases, and other complementary control actions may be abandoned or lessened as a result. While *Wolbachia* was assessed as a veterinary chemical product [36], the “causes more harm” formulation of risk is in line with the approach to potential risks from beneficial organisms presented in the Australian Biological Control Act 1984 [37]. This legislation is particularly intended for cases where there are conflicting interests concerned with releases that may be perceived as beneficial by some and not others [36], which is a situation that may often apply with GM insect applications. The scientists releasing *Wolbachia*-infected mosquitoes in Australia struggled to find national regulatory procedures that could be applied to their novel technology to ensure responsible oversight and approval. The risk assessors in that case have called for the regulatory process to be reviewed to ensure that it is appropriate for both proponents and regulators [36].

An overarching set of principles and standards for the safe rearing and intentional release of any insects, including GM insects, would ensure that the appropriate science is applied in all cases, a common level of risk assessment is conducted, and proportionate risk management is undertaken where risks are accepted. The “causes more harm” test may be a useful common denominator in achieving this, and the broad experience of mass releases of other species and technologies should inform the process.
